# Supplementary material for: Exploring Toxin Genes of Myanmar Russell’s Viper, Daboia siamensis, through De Novo Venom Gland Transcriptomics
Source: Toxins (Basel). 2023 Apr 26;15(5):309. doi: 10.3390/toxins15050309 (PMC10223203; doi:10.3390/toxins15050309)
Supplement: Supplementary file 1 [file toxins-15-00309-s001.zip › Table S3.pdf]

Supplementary Table S3. Transcripts with snake venom C-type lectin (CTL) annotations

Subtype.	Contig ID	Top Blast Hit Description	Accession	Species	Length	TPM value
I	c4038_g1_i1_M_PL	C-type lectin 1	Q6T7B7	*Bitis gabonica*	154	310.71
I	c373_g1_i2_F_PL	C-type lectin 1	Q6T7B7	*Bitis gabonica*	147	103.28
I	c373_g1_i1_F_PL	C-type lectin 1	Q6T7B7	*Bitis gabonica*	151	106.25
II	c20336_g1_i2_M_PL	p31 beta subunit	K9JDF6	*Daboia siamensis*	130	3756.69
II	c11770_g3_i8_F_PL	p31 beta subunit	K9JDF6	*Daboia siamensis*	145	1410.86
III	c20336_g1_i7_M_PL	RVV-X light chain 1	A0A2H4Z2X7	*Daboia siamensis*	151	8900.00
III	c11770_g3_i3_F_PL	RVV-X light chain 1	A0A2H4Z2X7	*Daboia siamensis*	141	3873.48
IV	c20336_g2_i1_M_FL	p68 alpha subunit	K9JBV0	*Daboia siamensis*	194	8064.14
IV	c20336_g2_i2_M_FL	p68 alpha subunit	K9JBV0	*Daboia siamensis*	159	8444.91
IV	c11770_g3_i5_F_PL	Snacle A15	B4XSZ0.1	*Macrovipera lebetina*	221	5880.20
V	c20336_g1_i8_M_PL	Snaclec dabocetin subunit alpha	Q38L02	*Daboia siamensis*	190	19785.34
V	c11770_g3_i6_F_PL	Snaclec dabocetin subunit alpha	Q38L02	*Daboia siamensis*	149	9149.85
V	c6062_g1_i1_M_PL	Snaclec A12	B4XSY7	*Macrovipera lebetina*	110	7503.30
V	c11770_g3_i7_F_PL	Snaclec A12	B4XSY7	*Microvipera lebetina*	149	1759.70
VI	c20336_g1_i9_M_PL	Factor X activator light chain 2	K9JDJ1	*Daboia siamensis*	163	8247.86
VI	c11770_g3_i4_F_PL	Factor X activator light chain 2	K9JDJ1	*Daboia siamensis*	153	3712.51
VII	c20336_g1_i3_M_PL	p68 beta subunit	K9JCR4	*Daboia russelii limitis*	188	7182.11
VII	c20336_g1_i4_M_PL	p68 beta subunit	K9JCR4	*Daboia russelii limitis*	159	7264.55
VII	c20336_g1_i1_M_FL	Snaclec 7	Q4PRC6	*Daboia siamensis*	171	6098.68
VII	c20336_g1_i6_M_FL	Snaclec 7	Q4PRC6	*Daboia siamensis*	190	7182.11
VII	c11770_g3_i2_F_PL	Snaclec 7	Q4PRC6	*Daboia siamensis*	145	3133.30
VII	c20336_g1_i5_M_PL	Snaclec-7	A0A1J0CZM7	*Vipera ammodytes ammodytes*	128	1692.90
VII	c11770_g3_i1_F_PL	Snaclec 7	Q4PRC6	*Daboia siamensis*	145	3235.51
