# Supplementary material for: Exploring Toxin Genes of Myanmar Russell’s Viper, Daboia siamensis, through De Novo Venom Gland Transcriptomics
Source: Toxins (Basel). 2023 Apr 26;15(5):309. doi: 10.3390/toxins15050309 (PMC10223203; doi:10.3390/toxins15050309)
Supplement: Supplementary file 1 [file toxins-15-00309-s001.zip › Table S4.pdf]

Supplementary Table S4. QUAST reports of assembled transcriptomes by two different assemblers

Assembly tools	Trinity		CLC Genomics	
Samples	Male	Female	Male	Female
Total number of contigs	33,106	20,248	23,791	16,930
Largest contig (bp)	31,422	17,302	29,849	15,013
Total length (bp)	58,894,508	29,920,371	39,721,466	23,760,199
N50	2,516	1,935	2,446	1,814
Mismatches (#N's per 100kbp)	0.00	0.00	144.97	96.38
