# Supplementary material for: Exploring Toxin Genes of Myanmar Russell’s Viper, Daboia siamensis, through De Novo Venom Gland Transcriptomics
Source: Toxins (Basel). 2023 Apr 26;15(5):309. doi: 10.3390/toxins15050309 (PMC10223203; doi:10.3390/toxins15050309)
Supplement: Supplementary file 1 [file toxins-15-00309-s001.zip › toxins-2207666-supplementary.pdf]

Supplementary Materials

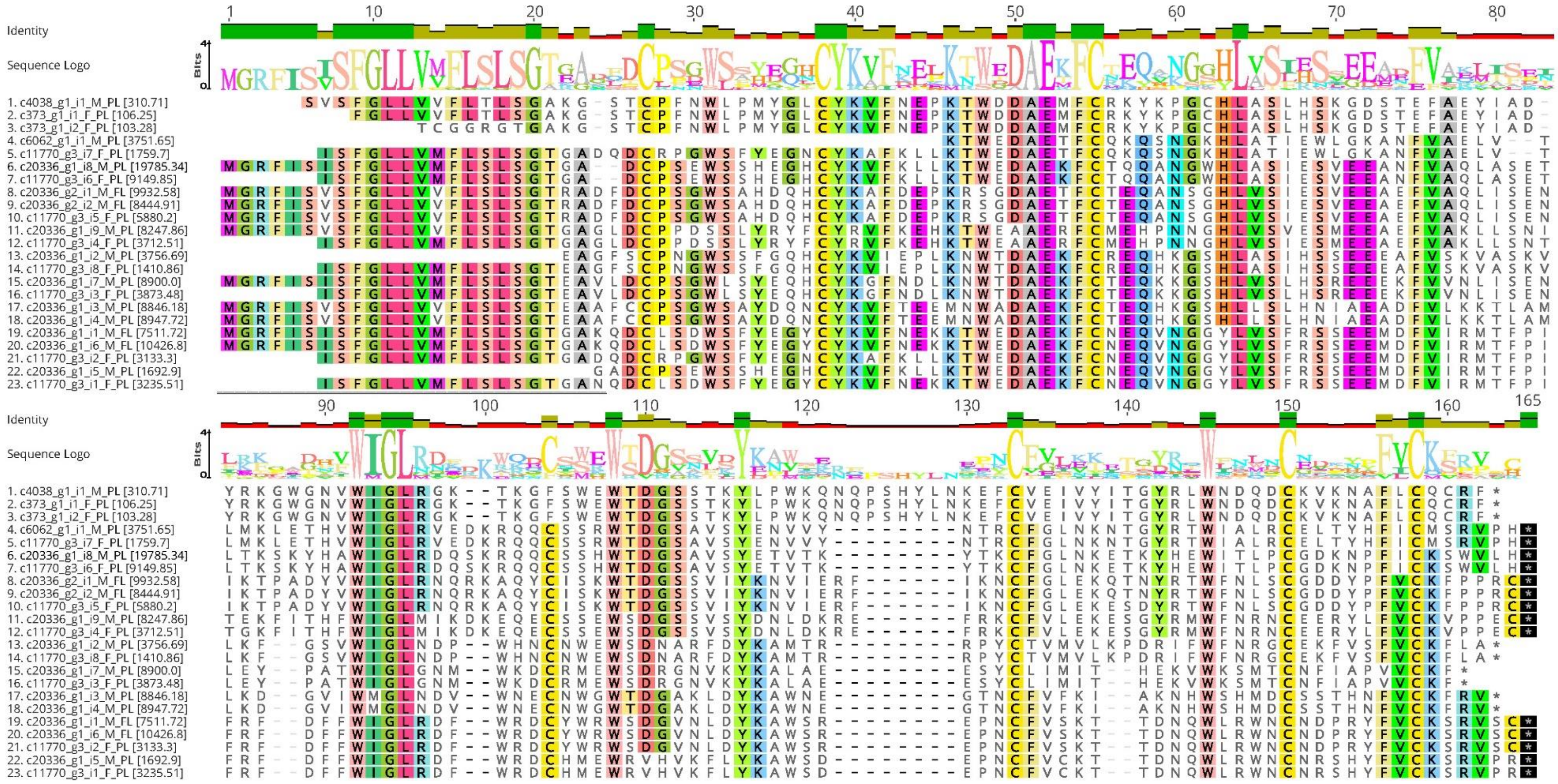

Figure S1. Clustal Omega sequence alignment of the polypeptides encoded by 23 transcripts candidate C-type lectins from Myanmar *D. siamensis*. Signal peptide was underlined, with conserved residues (>50%) highlighted throughout the alignment. Six of the cysteine residues were highly conserved, with each protein having typically 8 cysteine residues. The CTL conserved motif WIGL was highly conserved (position 92-95). The program Geneious Prime 2023. 0.2 was used to create this alignment, with SeqLogo [1] to highlight highly conserved amino acid residues. Expression values (TPM) were included in brackets following sequence name.

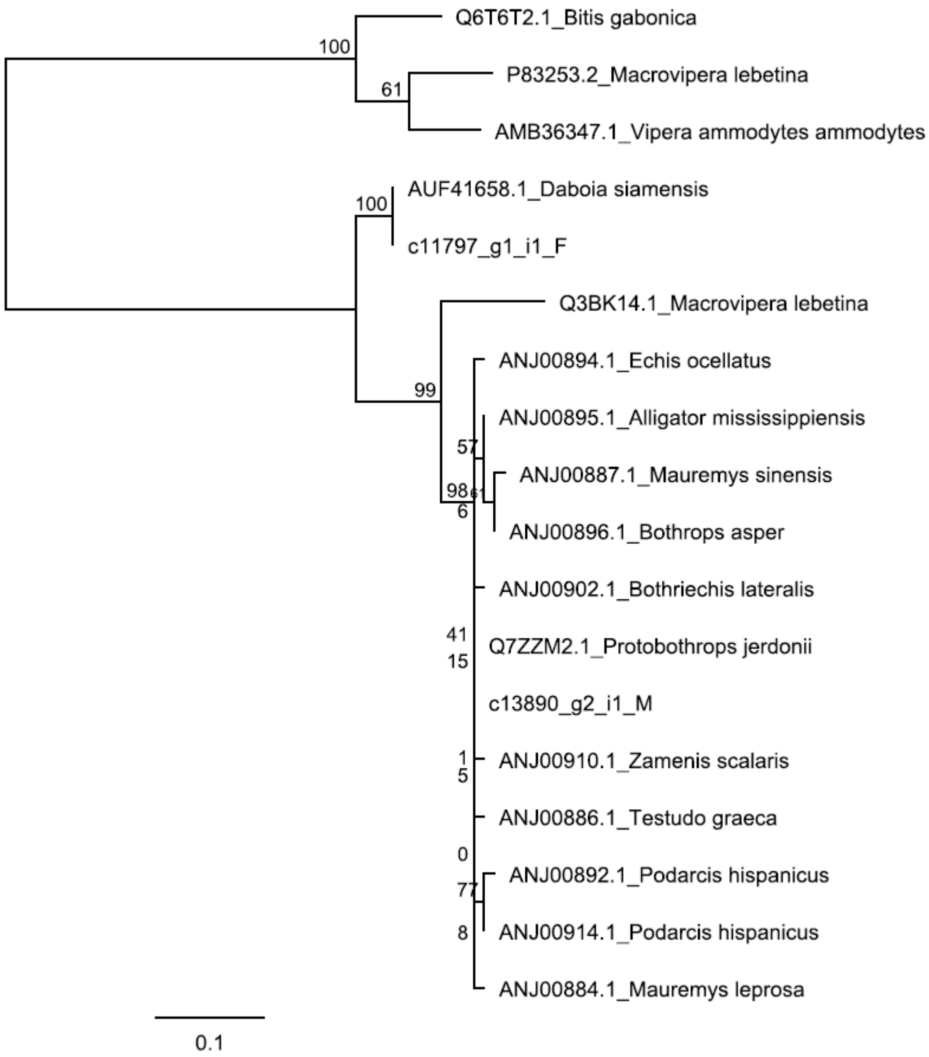

Figure S2. The gene tree analysis of the disintegrins from Myanmar *D. siamensis*, Dis1a (c13890_g2_i1_M) and Dis1b (c11797_g1_i1_F), with disintegrins from other species.

```
RVV_Vγ                 ----------MVLIKVLANLLVLQLSYAQKSSELVVGGDECNINEHPFLVALYTSASSTI   50
c20548_g3_i4_M_FL      ----------MVLIRVLANLLVLQLSYAQKSSELVVGGDECNINEHPFLVALYTSASSTI   50
c11820_g1_i1_F_PL      ----------MVLIRVLANLLVLQLSYAQKSSELVVGGDECNINEHPFLVALYTSASSTI   50
c11820_g1_i3_F_PL      MNSLPCRVEAMVLIRVLANLLVLQLSYAQKSSELVVGGDECNINEHPFLVALYTSASSTI   60
c11820_g1_i4_F_PL      MNSLPCRVEAMVLIRVLANLLVLQLSYAQKSSELVVGGDECNINEHPFLVALYTSASSTI   60
                                 ****:*********************************************

RVV_Vγ                 HCAGALINREWVLTAAHCDRRNIRIKLGMHSKNIRNEDEQIRVPRGKYFCLNTKFPNGLD   110
c20548_g3_i4_M_FL      HCAGALINREWVLTAAHCDRRNIRIKLGMHSKNIRNEDEQIRVPRGKYFCLNTKFPNGLD   110
c11820_g1_i1_F_PL      HCAGALINREWVLTAAHCDRRNIRIKLGMHSKNIRNEDEQIRVPRGKYFCLNTKFPNGLD   110
c11820_g1_i3_F_PL      HCAGALINREWVLTAAHCDRRNIRIKLGMHSKNIRNEDEQIRVPRGKYFCLNTKFPNGLD   120
c11820_g1_i4_F_PL      HCAGALINREWVLTAAHCDRRNIRIKLGMHSKNIRNEDEQIRVPRGKYFCLNTKFPNGLD   120
                       ************************************************************

RVV_Vγ                 KDIMLIRLRRPVTYSTHIAPVSLPSRSRGVGSRCRIMGWGKISTTEDTYPDVPHCTNIFI   170
c20548_g3_i4_M_FL      KDIMLIRLRRPVTYSTHIAPVSLPSRSRGVGSRCRIMGWGKIST--DTYPDVPHCTNIFI   168
c11820_g1_i1_F_PL      KDIMLIRLRRPVTYSTHIAPVSLPSRSRGVGSRCRIMGWGKIST--DTYPDVPHCTNIFI   168
c11820_g1_i3_F_PL      KDIMLIRLRRPVTYSTHIAPVSLPSRSRGVGSRCRIMGWGKIST--DTYPDVPHCTNIFI   178
c11820_g1_i4_F_PL      KDIMLIRLRRPVTYSTHIAPVSLPSRSRGVGSRCRIMGWGKIST--DTYPDVPHCTNIFI   178
                       ********************************************  **************

RVV_Vγ                 VKHKWCEPLYPWVPADSRTLCAGILKGGRDTCHGDSGGPLICNGEMHGIVAGGSEPCGQH   230
c20548_g3_i4_M_FL      VKHKWCEPLYPWVPADSRTLCAGILKGGRDTCHGDSGGPLICNGEMHGIVAGGSEPCGQH   228
c11820_g1_i1_F_PL      VKHKWCEPLYPWVPADSRTLCAGILKGGRDTCHGDSGGPLICNGEMHGIVAGGSEPCGQH   228
c11820_g1_i3_F_PL      VKHKWCEPLYPWVPADSRTLCAGILKGGRDTCHGDSGGPLICNGEMHGIVAGGSEPCGQH   238
c11820_g1_i4_F_PL      VKHKWCEPLYPWVPADSRTLCAGILKGGRDTCHGDSGGPLICNGEMHGIVAGGSEPCGQH   238
                       ************************************************************

RVV_Vγ                 LKPAVYTKVFDYNNWIQSIIAGNRTVTCPP-    260
c20548_g3_i4_M_FL      LKPAVYTKVFDYNNWIQSIIAGNRTVTCPP*    258
c11820_g1_i1_F_PL      LKPAVYTKVFDYN------------------    241
c11820_g1_i3_F_PL      LKPAVYTKVFDYN------------------    251
c11820_g1_i4_F_PL      LKPAVYTKVFDYN------------------    251
                       *************
```

Figure S3. Sequence alignment of RVV-V_{γ} from *Daboia siamensis* and deduced amino acid sequences of transcripts from the transcriptomes. Signal peptide is single-underlined. Catalytic triad His57, Asp102 and Ser195 are highlighted in yellow. Cysteine residues for 6 disulfide bridge are highlighted in green. (*) fully conserved residues; (:) strongly similar residues; (.) weakly similar residues.

```
c13118_g1_i1_M_FL      MAAYLLAVAILFCIQGWPSGTVQGQVRPFPDVYQRSACQTRETLVSILQEHPDEISDIFR   60
c22505_g1_i1_F_FL      MAAYLLAVAILFCIQGWPSGTVQGQVRPFPDVYQRSACQTRETLVSILQEHPDEISDIFR   60
VR_1                   MAAYLLAVAILFCIQGWPSGTVQGQVRPFLDVYERSACQTRETLVSILQEHPDEISDIFR   60
                       ***************************** ***:**************************

c13118_g1_i1_M_FL      PSCVAVLRCSGCCTDESMKCTPVGKHTADIQIMRMNPRTHSSKMEVMKFMEHTACECRPR   120
c22505_g1_i1_F_FL      PSCVAVLRCSGCCTDESMKCTPVGKHTADIQIMRMNPRTHSSKMEVMKFMEHTACECRPR   120
VR_1                   PSCVAVLRCSGCCTDESMKCTPVGKHTADIQIMRMNPRTHSSKMEVMKFMEHTACECRPR   120
                       ************************************************************

c13118_g1_i1_M_FL      WKQGEPEGPKEPRRGE*------- 136
c22505_g1_i1_F_FL      WKQGEPEGPKEPRRGE*------- 136
VR_1                   WKQGEPEGPKEPRRGGVRAKFPFD 144
                       ***************
```

Figure S4. Sequence alignment of deduced amino acid sequences of VEGF transcripts with VR-1 from *D. russelii* (P67861). Signal peptide is single-underlined. Cysteine residues are in yellow color. (*) fully conserved residues; (:) strongly similar residues; (.) weakly similar residues.

```
c11787_g15_i2_F_PL      ------------------------------------------------------------ 0
DrLAO                   MNVFFMFSLLFLATLGSCADDKNPLEECFREDDYEEFLEIAKNGLKKTSNPKHIVIVGAG 60
c17343_g1_i1_M_FL       MNVFFMFSLLFLATLGSCADDKNPLEECFREDDYEEFLEIAKNGLKKTSNPKHIVVVGAG 60
c11787_g15_i1_F_FL      MNVFFMFSLLFLATLGSCADDKNPLEECFREDDYEEFLEIAKNGLKKTSNPKHIVVVGAG 60

c11787_g15_i2_F_PL      -------------MSSVTVLEASERPGGRVRTHRNVKEGWYANLGPMRVPEKHRIIREYI 47
DrLAO                   MSGLSAAYVLAGAGHKVTVLEASERPGGRVRTHRNVKEGWYANLGPMRVPEKHRIIREYI 120
c17343_g1_i1_M_FL       MSGLSAAYVLAGAGHKVTVLEASERPGGRVRTHRNVKEGWYANLGPMRVPEKHRIIREYI 120
c11787_g15_i1_F_FL      MSGLSAAYVLAGAGHKVTVLEASERPGGRVRTHRNVKEGWYANLGPMRVPEKHRIIREYI 120
                                       .********************************************

c11787_g15_i2_F_PL      RKFGLKLNEFVQETENGWYFIKNIRKRVGEVKKDPGLLKYPVKPSEAGKSAGQLYQESLG 107
DrLAO                   RKFGLKLNEFVQETENGWYFIKNIRKRVGEVKKDPGLLKYPVKPSEAGKSAGQLYQESLG 180
c17343_g1_i1_M_FL       RKFGLKLNEFVQETENGWYFIKNIRKRVGEVKKDPGLLKYPVKPSEAGKSAGQLYQESLG 180
c11787_g15_i1_F_FL      RKFGLKLNEFVQETENGWYFIKNIRKRVGEVKKDPGLLKYPVKPSEAGKSAGQLYQESLG 180
                        ************************************************************

c11787_g15_i2_F_PL      KAVEELKRTNCSYILNKYDTYSTKEYLIKEGNLSPGAVDMIGDLLNEDSGYYVSFIESLK 167
DrLAO                   KAVEELKRTNCSYILNKYDTYSTKEYLIKEGNLSPGAVDMIGDLLNEDSGYYVSFIESLK 240
c17343_g1_i1_M_FL       KAVEELKRTNCSYILNKYDTYSTKEYLIKEGNLSPGAVDMIGDLLNEDSGYYVSFIESLK 240
c11787_g15_i1_F_FL      KAVEELKRTNCSYILNKYDTYSTKEYLIKEGNLSPGAVDMIGDLLNEDSGYYVSFIESLK 240
                        ************************************************************

c11787_g15_i2_F_PL      HDDIFAYEKRFDEIVGGMDQLPTSMYRAIEESVRFKARVIKIQQNAEKVTVTYQTTQKNL 227
DrLAO                   HDDIFAYEKRFDEIVGGMDQLPTSMYRAIEESVHFKARVIKIQQNAEKVTVTYQTTQKNL 300
c17343_g1_i1_M_FL       HDDIFAYEKRFDEIVGGMDQLPTSMYRAIEESVRFKARVIKIQQNAEKVTVTYQTTQKNL 300
c11787_g15_i1_F_FL      HDDIFAYEKRFDEIVGGMDQLPTSMYRAIEESVRFKARVIKIQQNAEKVTVTYQTTQKNL 300
                        *********************************:**************************

c11787_g15_i2_F_PL      LLETVDYVIVCTTSRAARRITFKPPLPPKKAHALRSVHYRSGTKIFLTCTKKFWEDDGIQ 287
DrLAO                   LLETADYVIVCTTSRAARRITFKPPLPPKKAHALRSVHYRSGTKIFLTCTKKFWEDDGIQ 360
c17343_g1_i1_M_FL       LLETVDYVIVCTTSRAARRITFKPPLPPKKAHALRSVHYRSGTKIFLTCTKKFWEDDGIQ 360
c11787_g15_i1_F_FL      LLETVDYVIVCTTSRAARRITFKPPLPPKKAHALRSVHYRSGTKIFLTCTKKFWEDDGIQ 360
                        ****.*******************************************************

c11787_g15_i2_F_PL      GGKSTTDLPSRFIYYPNHNFTTGVGVIIAYGIGDDANFFQALNLNECADIVFNDLSSIHQ 347
DrLAO                   GGKSTTDLPSRFIYYPNHNFTTGVGVIIAYGIGDDANFFQALNLNECADIVFNDLSSIHQ 420
c17343_g1_i1_M_FL       GGKSTTDLPSRFIYYPNHNFTTGVGVIIAYGIGDDANFFQALNLNECADIVFNDLSSIHQ 420
c11787_g15_i1_F_FL      GGKSTTDLPSRFIYYPNHNFTTGVGVIIAYGIGDDANFFQALNLNECADIVFNDLSSIHQ 420
                        ************************************************************

c11787_g15_i2_F_PL      LPKKDLQTFCYPSIIQKWSLDKYAMGAITTFTPYQFQHFSEALTAPVGRIFFAGEYTANA 407
DrLAO                   LPKKDLQTFCYPSIIQKWSLDKYAMGAITTFTPYQFQHFSEALTAPVGRIFFAGEYTANA 480
c17343_g1_i1_M_FL       LPKKDLQTFCYPSIIQKWSLDKYAMGAITTFTPYQFQHFSEALTAPVGRIFFAGEYTANA 480
c11787_g15_i1_F_FL      LPKKDLQTFCYPSIIQKWSLDKYAMGAITTFTPYQFQHFSEALTAPVGRIFFAGEYTANA 480
                        ************************************************************

c11787_g15_i2_F_PL      HGWIDSTIKSGLTAARDVNRASEL*         431
DrLAO                   HGWIDSTIKSGLTAARDVNRASEL-         504
c17343_g1_i1_M_FL       HGWIDSTIKSGLTAARDVNRASEL*         504
c11787_g15_i1_F_FL      HGWIDSTIKSGLTAARDVNRASEL*         504
                        ************************
```

Figure S5. Sequence alignment of DrLAO (*Daboia russelii*, **G8XQX1.1**) with 3 deduced LAAO transcripts obtained from Myanmar Russell's viper transcriptome. Conserved FAD-binding motif and Ile[1]-Hse[11] fragment is highlighted in green. Three potential N-glycosylation sites are underlined. Cysteine residues making disulfide bridge are highlighted in blue. Key residues in catalytic site are highlighted in yellow. (*) fully conserved residues; (:) strongly similar residues; (.) weakly similar residues.

```
c55720_g1_i1_M      MIAFIVLPILAAVLQQSSGSVDFDSESPRKPEIQNEIVDLHNSLRRSVTPTASNMLKMEW      60
c17345_g1_i1_F      MIAFIVLPILAAVLQQSSGSVDFDSESPRKPEIQNEIVDLHNSLRRSVTPTASNMLKMEW      60
Dr_CRPK             MIAFIVLPILAAVLQQSSGSVDFDSESPRRPEIQNEIVDLHNSLRRSVTPTASNMLKMEW      60
                    *****************************:******************************

c55720_g1_i1_M      YPEAAANAERWAFRCILNHSPYNSRVIGGIKCGENIYMSPYPMKWTAIIHEWHDEKKDFV      120
c17345_g1_i1_F      YPEAAANAERWAFRCILNHSPYNSRVIGGIKCGENIYMSPYPMKWTAIIHEWHDEKKDFV      120
Dr_CRPK             YPEAAANAERWAFRCILNHSPYNSRVIGGIKCGENIYMSPYPMKWTAIIHEWHKEKKDFV      120
                    *****************************************************.******

c55720_g1_i1_M      YGQGASPANAVVGHYTQVVWYKSYRSGCAAAYCPSSEYNYFYVCQYCPAGNIIGKIATPY      180
c17345_g1_i1_F      YGQGASPANAVVGHYTQVVWYKSYRSGCAAAYCPSSEYNYFYVCQYCPAGNIIGKIATPY      180
Dr_CRPK             YGQGASPANAVVGHYTQIVWYKSYRSGCAAAYCPSSEYNYFYVCQYCPAGNIIGKIATPY      180
                    *****************:******************************************

c55720_g1_i1_M      TSGPPCGDCPSACDNGLCTNPCSHHDDFTNCKDLVKQGCHSNYLKTKCPASCFCHNEII       239
c17345_g1_i1_F      TSGPPCGDCPSACDNGLCTNPCSHHDDFTNCKDLVKQGCHSNYLKTKCPASCFCHNEII       239
Dr_CRPK             TSGPPCGDCPSACDNGLCTNPCSHHDEFTNCKDLVKQGCHSNYLKTKCPASCFCHNEII       239
                    *************************:*********************************
```

Figure S6. Sequence alignment of deduced amino acid sequences of CRISP transcripts from the transcriptomes with cysteine-rich secretory protein Dr-CRPK from Taiwan Russell's viper (ACE73567). Signal peptide is single-underlined. Cysteine residues are in pink color. The two Zn^{2+} binding motifs are highlighted in yellow and blue. (*) fully conserved residues; (:) strongly similar residues; (.) weakly similar residues.

References

1. Bembom, O.; Ivanek, R. seqLogo: Sequence logos for DNA sequence alignments. R pakage version 1.64.0. Available online: https://bioconductor.org/packages/release/bioc/html/seqLogo.html (accessed on 26 March 2022).
